# Fish Assemblages of Mediterranean Marine Caves

**DOI:** 10.1371/journal.pone.0122632

**Published:** 2015-04-13

**Authors:** Simona Bussotti, Antonio Di Franco, Patrice Francour, Paolo Guidetti

**Affiliations:** 1 Université Nice Sophia Antipolis, Faculté des Sciences, EA 4228 ECOMERS, Nice, France; 2 CoNISMa (National Interuniversity Consortium of Marine Sciences), Rome, Italy; Università di Genova, ITALY

## Abstract

Fish assemblages associated with 14 marine caves and adjacent external rocky reefs were investigated at four Marine Protected Areas (MPAs) along the coasts of Italy. Within the caves sampling was carried out in different sub-habitats: walls, ceilings, bottoms and ends of caves. On the whole, 38 species were recorded inside the 14 caves investigated. Eighteen species were exclusively found inside the caves: they were mainly represented by speleophilic (i.e. species preferentially or exclusively inhabiting caves) gobids (e.g. *Didogobius splechtnai*) and nocturnal species (e.g. *Conger conger*). Forty-one species were censused outside, 20 of which were shared with cave habitats. *Apogon imberbis* was the most common fish found in all 14 caves investigated, followed by *Thorogobius ephippiatus* (recorded in 13 caves), and *Diplodus vulgaris* and *Scorpaena notata* (both censused in 12 caves). Distinct fish assemblages were found between external rocky reefs and the different cave sub-habitats. New data on the distribution of some speleophilic gobids were collected, showing the existence of a pool of species shared by marine caves on a large scale (i.e. hundreds of km). Considering the uniqueness of cave fishes (18 exclusive species and different assemblage structures), the inclusion of marine caves among the habitats routinely investigated for fish biodiversity monitoring could facilitate the achievement of more comprehensive inventories. Due to their contribution to local species diversity and the shelter they provide to species valuable for conservation, marine caves should be prioritized for their inclusion not only within future MPAs through the Mediterranean Sea, but also into larger management spatial planning.

## Introduction

Along the coasts of the Mediterranean Sea, especially in rocky limestone areas subject to karstic dissolution, submerged marine caves are widespread and mostly unexplored natural habitats [1]. Because of the intrinsic variability and complexity of submerged marine cave systems, it is difficult to formally define them. In the Mediterranean context, some authors [2], [3] provided an operative and general definition: a “marine cave” is a cavity of various origin, completely or partially submerged, characterised by 1) a ratio between ‘overall internal volume/surface of the opening’ higher than 1, and 2) a width of the opening that should not exceed the mean width inside.

Due to their special ecological conditions inside, e.g. in terms of gradients of light intensity, water motion and distribution of trophic resources [2], [3], marine caves in the Mediterranean host unique and fragile biological communities (see e.g. [1], [4], [5–7]). Considering that benthic organisms living in coastal rocky habitats outside the caves usually compete for space occupation [8], [9], the presence of caves can also be seen as increasing the availability of coastal rocky substrates (see [10]).

Several coastal fishes can be found inside marine caves in the Mediterranean Sea. They encompass 1) species exclusively associated with marine caves (i.e. speleophilic), 2) nocturnal species (normally remaining within refuges during the day but moving outside at night), 3) species typical of cryptic habitats (including caves but also relatively small holes, under stones and crevices in the bedrock outside the caves), and 4) nectobenthic species generally associated with rocky reefs that can be found occasionally within caves, often in their initial portions [2], [10–17]. Cryptic habitats, including marine caves, have also been reported to host lessepsian fishes (Red Sea and Indo-Pacific species that enter the Mediterranean through the Suez Canal). It is case, for instance, of the dusky sweeper, *Pempheris rhomboidea*, in the Eastern Mediterranean, whose taxonomic aspects have been recently elucidated in [18]. Along some stretches of Turkish coast (e.g. around Antalya, Mediterranean Sea; Guidetti, pers. obs., but see also [19]), *P*. *rhomboidea* occupies the same micro-habitats (i.e. small crevices, under stones and boulders) that *Apogon imberbis* (native fish) generally occupies in the western Mediterranean, where it is very common [20]. In the case of further spreading of *P*. *rhomboidea* westwards it is reasonable to hypothesize competition between the alien and the native fish for the occupation of caves and any other cryptic habitat [21].

All the above issues suggest that assessing the fish fauna of marine caves may have important implications for conservation and management, including the monitoring of the spreading of some potentially invasive species.

The available scientific literature dealing with fish inhabiting Mediterranean marine caves is mainly focused on micro-habitat requirements of small cryptic species (e.g. [13], [22–25]). Abel [11], first, defined the ‘cave habitat’ for fishes as any ‘hole’ able to host the body of a fish, thus relating the spatial extension of the ‘cave’ to the body size of fish. He also created the term “cave in cave” for the specific territories of the speleophilic *Microlipophrys nigriceps* inhabiting piddock holes (small holes produced by boring mollusks within caves). Zander [22] used the term ‘mesolithion’ to define the cavity systems characterized by a decrease of light and water movement, independently of cavity size. Patzner [24] distinguished different cryptic habitats for coastal fishes, i.e. cavities (with cavity depth/length smaller that height/width, and low light levels) and caves (whose depth/length always exceeds height/width of the entrance, scarcely lighted to completely dark, sometimes with bottoms covered with fine sediment).

More active swimming fishes, such as sea breams, mullets, small serranids, may inhabit marine caves. However, they have in general received little attention compared with small cryptic species. Similarly, macroscopic cave features potentially affecting presence and distribution of fish (e.g. topography, presence/absence of secondary openings, presence/absence of air chambers, freshwater inputs, wave exposure, dimensions and number of tunnels and/or chambers, rocky or muddy bottom, etc.) have seldom been quantitatively assessed. Arko-Pijevac *et al*. [14] noted that the bulk of studies carried out on Mediterranean fish cave assemblages were done mostly 1) on small-sized sedentary cryptic fishes, and 2) within caves of limited size (<10 m long) often without reporting specific information on cave dimensions, structure and variety of sub-habitat types inside (e.g. openings, obscure ends, ceiling).

A more exhaustive description of cave habitats should take into account a number of features and scales potentially relevant in structuring fish assemblages. However, morphological features of marine caves are extremely variable from cave to cave (see [26]). This may have a strong influence on the diversity and structure of fish assemblages hosted inside, and can make it quite difficult to apply standardized approaches (i.e. formal sampling designs) to collect field data.

In recent years some studies on fish assemblages associated with Mediterranean marine caves were done following guidelines that formally satisfied fundamental aspects of sampling design theory applied to ecological studies (see e.g. [27]), like spatial replication and comparisons of caves showing similar structures to assess patterns of spatio-temporal variability [10], [15], [16]. The adoption of similar approaches, however, implies that the selection of caves to be included in the study is restricted to caves with comparable morphology (e.g. depth, size and general shape), in order to avoid any confounding effect of uncontrolled factors. However, such a ‘forced’ selection of specific (i.e. comparable) types of caves excludes all caves that don’t fit the *a priori*-selected morphological criteria. Overweighting the need for more experimental rigor in order to get formally perfect data or to reply to specific questions, therefore, carries the risk to lose important pieces of information when the overall diversity of fish is to be captured. Cave structures and related ecological factors, in fact, may indeed greatly change from cave to cave having a variable morphology and other features sometimes difficult to formalize. Such a variability can be mirrored in the structure and diversity of associated fish assemblages (e.g. in terms of species composition and patterns of abundance). Assessing more exhaustive diversity patterns (using an approach that properly takes into consideration natural history issues [28]) can produce useful information, for example when fish assemblages associated with various habitats (including caves) are hosted inside Marine Protected Areas (hereafter MPAs). This is especially true, talking about caves, wherever MPAs are established in karstic regions where marine caves are numerous and variable in structure [5], [29]. So, in some cases, it could be preferable to carry out fish sampling inside marine caves that are structurally as variable as possible. This would maximize the capture of the overall variability and diversity (e.g. in terms of species present) in the associated fish fauna in a given stretch of coast or region.

Along the coasts of Italy (including the main islands of Sicily and Sardinia, and many other small archipelagos), many MPAs have been established in recent years, including in coastal areas rich in marine caves. Although marine caves are listed among the few marine habitats under protection for the European Union (EU Habitat Directive 92/43EEC) and they are becoming more and more popular for recreational diving (a serious threat for many fragile and shy organisms living inside), there are no specific rules or restrictions (except in a few MPAs). In recent years, a number of papers specifically emphasized the contribution of cave assemblages to overall coastal biodiversity (e.g. [[6], [7], [10], [30], [31])and/or assessed the detrimental effects of some human impacts on associated benthic communities (such as unregulated underwater activities; see [32], [33]). This type of information is necessary to implement proper conservation and management measures [34], [35].

The demand for more effective protection and management policies of marine caves in the Mediterranean Sea can be highly supported by evidencing their contribution to marine biodiversity. The information available on the diversity of the fish fauna hosted inside the caves is therefore crucial.

In this study, we thus evaluated 1) the variability in fish species richness and assemblage structure in 14 marine caves of different morphology located inside four Italian MPAs, and 2) the additional contribution of such environments to local fish diversity associated with rocky reefs.

## Methods

### Ethics statement

All the data collected in the present study have been gathered by using a non destructive methodology (commonly named as ‘Underwater Fish Visual Census’). No fish was collected, injured or manipulated. In addition, scientific divers adopted a proper behavior underwater in order to limit any disturbance into the caves investigated. The research, from a formal point of view, has been funded and committed by the Italian Ministry of the Environment, which is the state authority competent on MPAs in Italy. Funding and commitment implied the authorization to access the MPAs and run the non destructive sampling on fish. In addition, in the occasion of each sampling campaign at each MPA, specific authorizations have been asked and obtained by the management bodies, formally represented by the following MPAs Managers: Gianfranco Russino (Capo Caccia MPA), Salvatore Martello (Lampedusa MPA), Vincenzo Incontro (Plemmirio MPA) and Paolo D’Ambrosio (Porto Cesareo MPA). Permissions from MPAs (besides the one released by the competent Ministry that funded the project) have been obtained in the form of simple letters or emails, so there isn’t any associated authorization numbers or codes.

### Study locations

**Fig 1:**
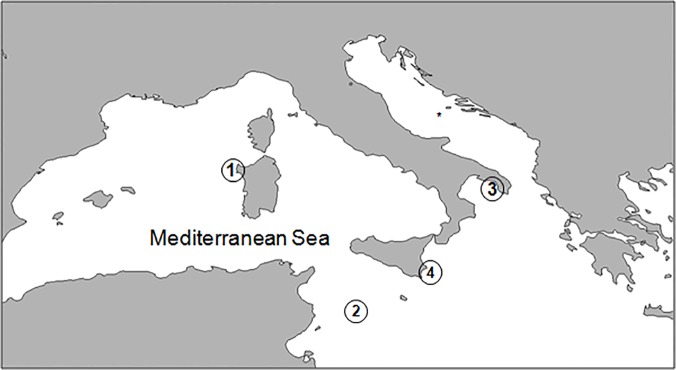
Location of the 4 MPAs hosting the 14 marine caves investigated in this study. 1, Capo Caccia; 2, Lampedusa; 3, Porto Cesareo; 4, Plemmirio.

Fish sampling was carried out in September and October 2009 in 14 submerged caves located in 4 Italian MPAs (Fig 1): 1) six caves at Capo Caccia (40° 33′ N, 8° 9′ E), Sardinia between ~5 and 18 m depth; 2) two caves at Lampedusa (35° 29' N—12° 36' E), Pelagie Islands, Sicily between ~10 and 20 m depth; 3) two caves at Porto Cesareo (40° 15' N—17° 53 E), Apulia between 8 and 10 m depth; 4) four caves at Plemmirio (37° 01' N—15° 19' E) near Siracusa, Sicily between ~18 and 30 m depth.

The investigated caves encompassed both blind caves (one entrance) and caves with several openings, that varied remarkably in terms of morphology, branching and overall extension, presence/absence of an underwater ceiling, depth and characteristics of the bottom (e.g. formed by rocks or muddy sediments, [36], [37]). A detailed description of the investigated marine caves is given in Table 1.

**Table 1:** Description of the main features of caves studied at the four Marine Protected Areas.

	CAVES													
	Capo Caccia						Lampedusa		Porto Cesareo		Plemmirio			
	B	C	F	G	N	T	TVI	TVII	Cor	X	Gy	Gr	LG	M
Total length (m)	36	30	50	70	200	55	57	35	50	20	65	55	20	30
Number of openings	1	2	2	1	6	1	3	2	1	1	1	2	1	1
Number of chambers	1	2	2	1	5	4	1	2	3	1	3	3	2	2
Substrate type	R/S	R/S	R	S	R/S	R	R	R/S	M/R	R	M	M	M	M
Air chambers	YES	YES	YES	YES	YES	YES	NO	YES	YES	NO	NO	NO	NO	NO
Accessible ends	YES	YES	YES	NO	NO	NO	NO	NO	NO	YES	NO	NO	NO	NO
Maximum depth (m)	10	10	12	9	30	12	20	16	17	10	25	20	25	30
Number of visual censuses	8	8	8	7	16	7	5	5	6	4	9	4	4	3

Cave abbreviations: B: Bisbe; C: Cabirol; F: Falco; G: Galatea; N: Nereo; T: Taleia; TVI: Taccio Vecchio 1; TVII: Taccio Vecchio 2; Cor: Corvine; X: Cave X; Gy: Gymnasium; Gr: Granchi; LG: Lingua del Gigante; M: Mazzare. Substrate type abbreviations: M: muddy; R: rocky; S: sandy.

### Sampling and data analyses

Fish abundance was estimated using a modified transect visual census method using SCUBA [10]. The scientists-divers who got the fish data within the caves investigated, received a specific training, as it is usually recommended. Marine caves, in fact, typically contain silt on the floor, which can easily be stirred up reducing visibility to zero and making it difficult if not impossible to locate the cave entrance (casualties are unfortunately not that rare). Transects were 8 to 60 m long and 2 m wide, as transect length was adapted to the shape and morphological discontinuities of different cave sectors. Any overlap between transects (which would have implied spatial dependence among replicates and a bias in the statistical analyses, see [27]) has been avoided. We have operatively defined as ‘sub-habitats’ the following sectors identified within the investigated caves: walls (coded as W), ceilings (C), bottoms (B). Four caves (i.e. Falco, Bisbe and Cabirol in the MPA of Capo Caccia and Cave X in the MPA of Porto Cesareo) presented easily accessible ends that were considered as a further sub-habitat type (E). The number of transects performed in each cave was also variable in relation to the extent and topography. Additional sampling was performed on rocky reef habitats outside each cave (codes as O) for comparison. Considering caves and rocky reefs outside the caves, a total of n = 94 visual censuses were performed in the present study (see Table 1 for detail about replication in each cave). With regard to the different sub-habitats, we performed n = 41 transects in W, n = 20 in C, n = 15 in B, n = 4 in E and, in addition, n = 14 in habitat O.

Due to the variable dimensions of transects, abundance values observed in the field were then converted to number of individuals/100 m^2^. Outside the caves, in agreement with standard methods on rocky reefs [38], visual censuses were carried out along 25 m long and 5 m wide transects. Densities were then converted to 100 m^2^.

Whole fish assemblage structures (i.e. in terms of species composition and relative abundances) were analyzed using 2-way unbalanced permutational multivariate analysis of variance (PERMANOVA; [39]) according to two factors: ‘caves’ (Ca), random with 14 levels, and ‘sub-habitat’ (SH), fixed with 5 levels (W, C, B, E, and O). Analyses were performed using the PRIMER v6 software [40], implemented with the add-on package permanova+ [41].

PERMANOVA analysis is capable of handling unbalanced multi-factorial experimental designs [41]. In order to avoid any potential bias due to the adoption of transects of different length (as specified above), prior to perform PERMANOVA, we checked for possible correlation among transect length and multivariate data and relevant single variables (i.e. species richness, total density). Specifically we used linear univariate/multivariate regression analyses (DISTLM). Multivariate regression was graphically represented by mean of distance-based redundancy analysis (dbRDA, [42]).

As no evidence of significant relationships was highlighted for all the variables considered (see S1 Fig, S2 Fig and S3 Fig. for supporting results), abundance values/100 m^2^ were adopted in PERMANOVA analysis.

To visualize multivariate patterns, non-metric multidimensional scaling (nMDS) ordinations were obtained from Bray-Curtis dissimilarity matrices calculated from log(x+1) transformed data. This transformation was selected to balance the contribution to overall abundances of schooling species (like *Apogon imberis*) and, therefore, reduce the weighting of abundant species and increase that of rarer species.

## Results

A total of 59 taxa was recorded in this study (S1 Table). Thirty-eight species were recorded inside the 14 investigated marine caves (Table 2). Twenty-eight, 18, 16 and 15 species were recorded in caves located within Capo Caccia, Porto Cesareo, Plemmirio and Lampedusa MPAs, respectively.

**Table 2:** Fish species recorded inside each marine cave at the four Marine Protected Areas.

	CAVES													
Family	Capo Caccia						Lampedusa		Porto Cesareo		Plemmirio			
*Species*	B	C	F	G	N	T	TVI	TVII	Cor	X	Gy	Gr	LG	M
Apogonidae														
* Apogon imberbis* ^b,c,e,w,o^	X	X	X	X	X	X	X	X	X	X	X	X	X	X
Blenniidae														
* * ***Microlipophrys nigriceps*** ^b,c,w^	X	X			X	X	X	X		X				
* * ***Parablennius rouxi*** ^w,o^					X									
* * ***Parablennius tentacularis*** ^w^										X				
* Parablennius zvonimiri* ^w,o^		X												
Bothidae														
* * ***Bothus podas*** ^b^	X													
Bythitidae														
* * ***Grammonus ater*** ^c,w^				X	X	X					X			
Carangidae														
* * ***Trachurus trachurus*** ^w^									X					
Congridae														
* * ***Conger conger*** ^c,w^			X								X	X		
Centracanthidae														
* Spicara maena* ^w,o^									X					
Dasyatidae														
* * ***Dasyatis centroura*** ^w^							X							
Gadidae														
* * ***Trisopterus capelanus*** ^w^					X									
Gobiidae														
* * ***Corcyrogobius liechtensteini*** ^c,w^	X	X	X		X	X	X	X	X	X	X		X	
* * ***Didogobius splechtnai*** ^e,w^					X		X			X				
* * ***Gammogobius steinitzi*** ^c,e,w^	X	X	X	X	X	X	X							
* * ***Gobius cruentatus*** ^w^											X			
* * ***Thorogobius ephippiatus*** ^b,c,e,w^	X	X	X	X	X	X		X	X	X	X	X	X	X
Labridae														
* Coris julis* ^b,e,w,o^	X				X	X			X					
Mullidae														
* Mullus surmuletus* ^b,e,w,o^	X		X								X	X		
Muraenidae														
* Muraena helena* ^w,o^					X									
Ophidiidae														
* * ***Ophidion barbatum*** ^b,w^					X									
Phycidae														
* * ***Phycis phycis*** ^**b,e,w**^		X		X	X									X
Pomacentridae														
* Chromis chromis* ^c,w,o^	X		X	X	X	X	X			X	X			
Scorpaenidae														
* Scorpaena maderensis* ^c,o1^										X				
* Scorpaena notata* ^b,c,e,w,o^	X	X	X	X	X	X	X		X	X	X	X	X	
* * ***Scorpaena scrofa*** ^*w*^											X		X	
Serranidae														
* * ***Anthias anthias*** ^b^												X		
* Epinephelus costae* ^w,o^							X							
* Epinephelus marginatus* ^w,o^	X				X		X							
* Serranus cabrilla* ^b,c,e,w,o^	X	X	X	X	X	X						X		
* Serranus scriba* ^c,w,o^		X				X	X	X	X	X	X	X	X	
Sciaenidae														
* * ***Sciaena umbra*** ^b,w^					X	X		X	X		X	X		X
Sparidae														
* Boops boops* ^w,o^									X					
* Diplodus annularis* ^w,o^			X		X									
* Diplodus puntazzo* ^w,o^			X											
* Diplodus sargus* ^w,o^	X	X	X						X					
* Diplodus vulgaris* ^b,e,w,o^	X	X	X	X	X	X	X	X	X		X	X		X
* Oblada melanura* ^b,w,o^		X	X	X	X	X		X	X					
Total	14	13	14	10	21	14	12	8	13	10	13	10	6	5

The letter beside the species name indicates the frequented cave sub-habitats and outside rocky reefs: b = bottom; c = ceiling; e = end of the cave; w = wall; o = outside. Species that were found exclusively inside caves are indicated in bold. Cave abbreviations: B: Bisbe; C: Cabirol; G: Galatea; F: Falco; N: Nereo; T: Taleia; TVI: Taccio Vecchio 1; TVII: Taccio Vecchio 2; Cor: Corvine; X: Grotta x; Gy: Gymnasium; Gr: Granchi; LG: Lingua del Gigante; M: Mazzare.

Eighteen species were exclusively found inside the caves (i.e. they were not recorded in the outside rocky reefs) and were represented mainly by speleophilic gobids (e.g. *Didogobius splechtnai*) and nocturnal predator species (e.g. *Conger conger*). Forty-one species were censused in the rocky reefs outside, 21 of which were exclusively found outside caves and 20 of which were shared with cave sub-habitats.

**Fig 2:**
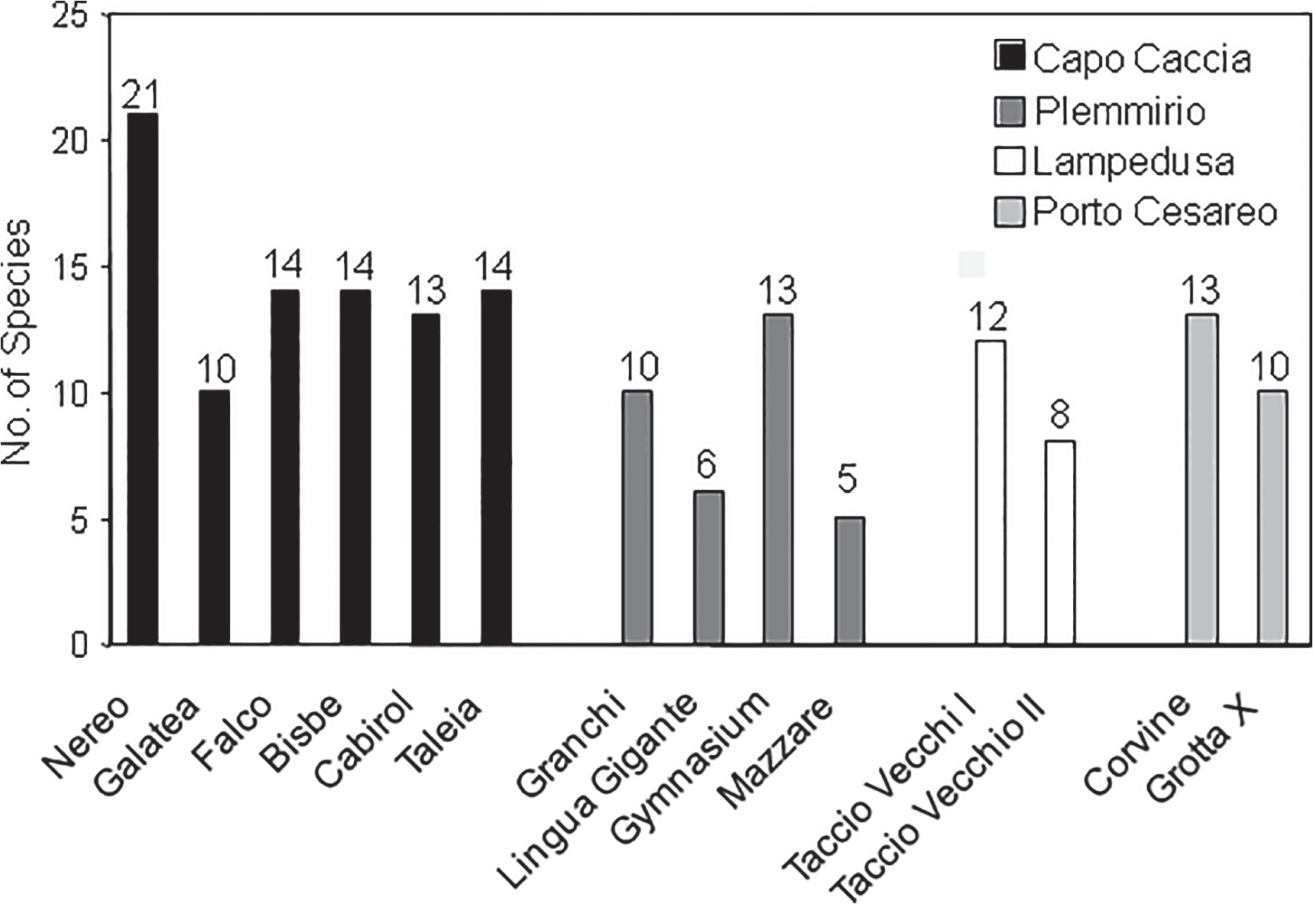
Number of the fish species recorded in the 14 marine caves investigated.

Considering cave by cave, the highest number of species (n = 21) was found inside the Nereo cave (the most extended cave among those investigated in this study) at Capo Caccia MPA and the lowest (n = 5) inside the Mazzare cave at Plemmirio MPA (Fig 2). A higher number of species was associated with W sub-habitat (n = 35) compared to B (n = 14), C (n = 12), or E sub-habitat (n = 10).

**Fig 3:**
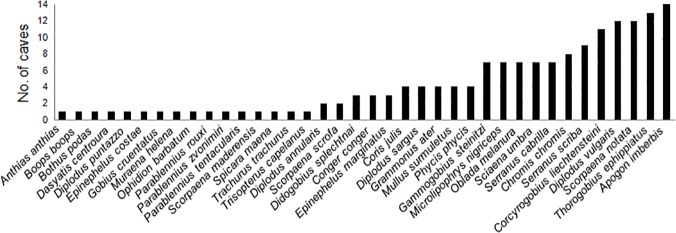
Frequency of fish species (expressed as number of caves where each fish has been recorded) at the 14 marine caves investigated.

In terms of frequency of occurrence, *Apogon imberbis* was the most frequent and widespread fish across the 14 caves investigated (Fig 3), followed by the speleophilic gobid *Thorogobius ephippiatus* found in 13 caves. *Diplodus vulgaris* and *Scorpaena notata* were found in 12 out of 14 caves, followed by *Corcyrogobius liechtensteini* censused in 11 caves. Other speleophilic gobids, like *Gammogobius steinitzi* and *Didogobius splechtnai*, were recorded in 7 and 4 caves, respectively, out of the 14 investigated. *Serranus scriba* and *Chromis chromis* have been censused in 9 and 8 caves, respectively. The remaining species were found in 7 or less marine caves out of 14 studied. The speleophilic fish *Grammonus ater* was quite rare: it was found in 4 out of 14 caves, at Capo Caccia and Plemmirio MPAs. It is worth noting the presence of several specimens of *Dasyatis centroura* inside the Taccio Vecchio 1 cave at Lampedusa.

PERMANOVA on fish assemblage structures associated with different caves and sub-habitats revealed a significant interaction between the two factors analyzed (Ca x SH; Table 3). Though both the main effects of the two factors investigated (‘caves’ and ‘sub-habitat’) were found to be highly significant, the output about the interaction Ca x HT suggests that there are differences in fish assemblage structures among sub-habitats that, however, are not coherent among caves.

**Table 3:** PERMANOVA based on Bray-Curtis dissimilarities of log_10_ (x+1) transformed data from 60 variables.

Source of variation	df	SS	MS	Pseudo-F	P(perm)	Unique
						perms
Cave = Ca	11	23192	2108.3	1.7835	0.003	998
Sub-habitat = SH	4	64995	16249.0	8.1515	0.001	999
Ca x SH	29	62374	2150.8	1.8194	0.001	997
Res	38	44921	1182.1			
Total	82	2.13E5				

**Fig 4:**
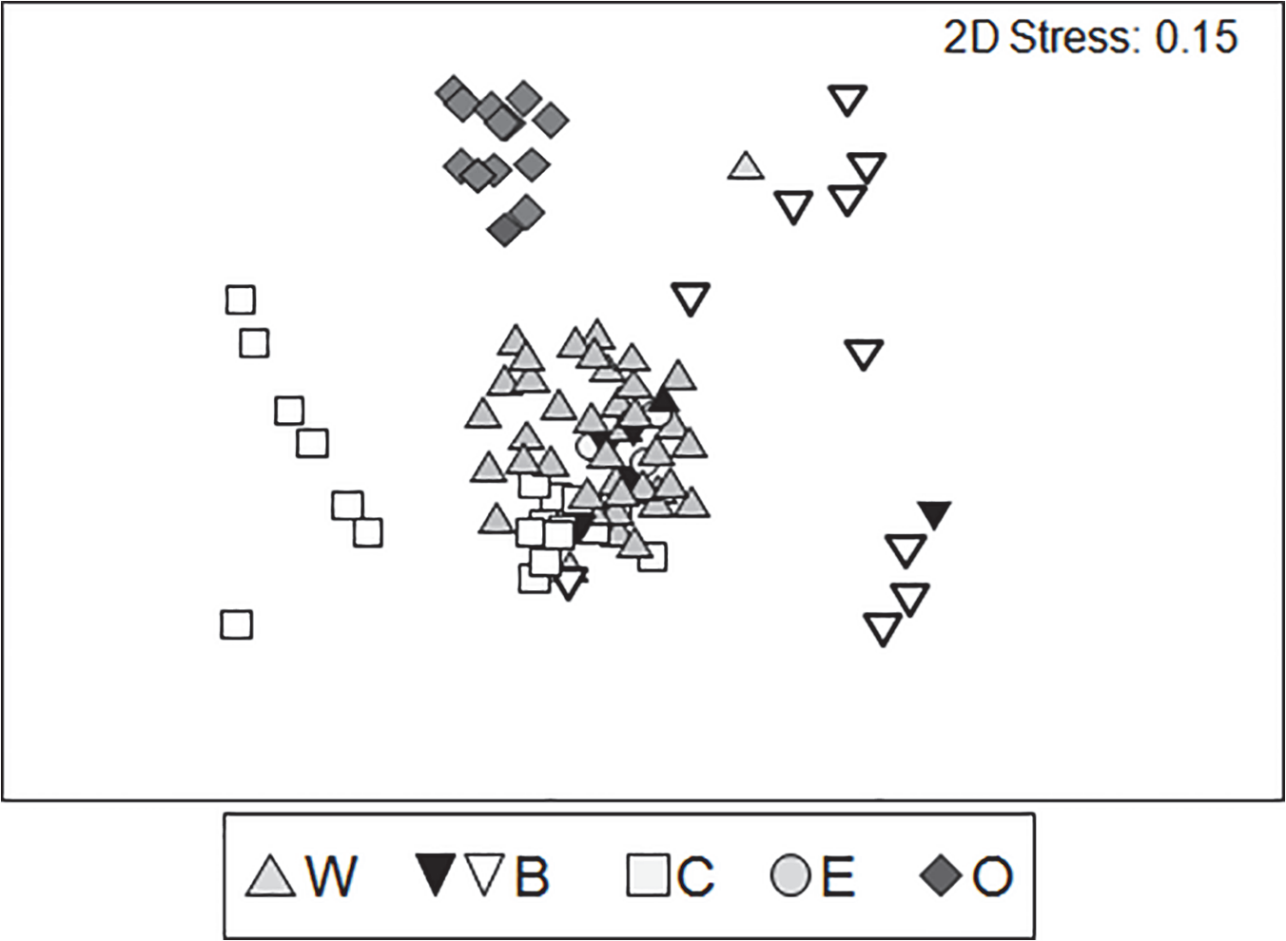
Non-metric MDS plots of individual replicates (i.e. individual visual census transects) of fish assemblages among the sub-habitat types present at all the 14 caves investigated. W: wall; B: bottom (white triangles = presence of sand/mud; dark triangles = ‘pure’ rocky bottoms): C: ceiling; E: end of the cave; O: rocky reefs outside the cave.

The nMDS plot of samples (i.e. showing all visual census transects; Fig 4) clearly shows that 1) fish assemblages associated with O separate out from those generally associated with caves; 2) fish assemblages associated with W are strongly aggregated (except for one single replicate census where *Apogon imberbis*, generally very abundant, was absent); 3) fish assemblages associated with C were in some cases similar to those associated with other rocky sub-habitats (e.g., W); 4) assemblages associated with bottoms characterized by a sandy/muddy component separate out from those associated with rocky floors (see Table 1 for details about bottom types in the various caves); 5) fish assemblages recorded in the final cave portions and on the rocky bottom sub-habitats fell within the cloud of points representing the assemblages observed on W.

## Discussion

To the best of our knowledge the number of species reported in this study is the highest ever reported from cave habitats in the Mediterranean Sea. According to Bussotti and Guidetti [10], we found that cryptic and shy species (e.g. *Apogon imberbis*, *Scorpaena notata*, *Thorogobius ephippiatus*, *Grammonus ater*) were mostly or exclusively found inside the caves. Some of these species were in almost all the caves investigated, with *Apogon imberbis* being reported everywhere. This result leads us to consider, especially taking into account the large spatial scale explored in this study, that a subset of fish species censused here could actually represent a pool typical of Mediterranean caves.

Other species are reported to be associated with caves, e.g. some Gobiidae such as *Odondebuenia balearica*, *Speleogobius trigloides* or *Vanneaugobius dollfusi* (P. Francour unpublished data; [23] and references therein). In the caves investigated here, the above-mentioned species were not recorded. This study, however, was just based on visual inspection, while destructive investigations using anesthetic [23] could allow to get more exhaustive species lists of cryptic fishes associated with marine caves.

For some speleophilic gobies, we found the same preference for cave sub-habitats as reported by other authors. *Corcyrogobius liechtensteini* and *Gammogobius steinitzi* were observed in cave walls and ceilings [13], [14], while *Didogobius splechtnai* was observed close to the bare rocky walls where this fish uses the small holes as hiding places [13].

**Fig 5:**
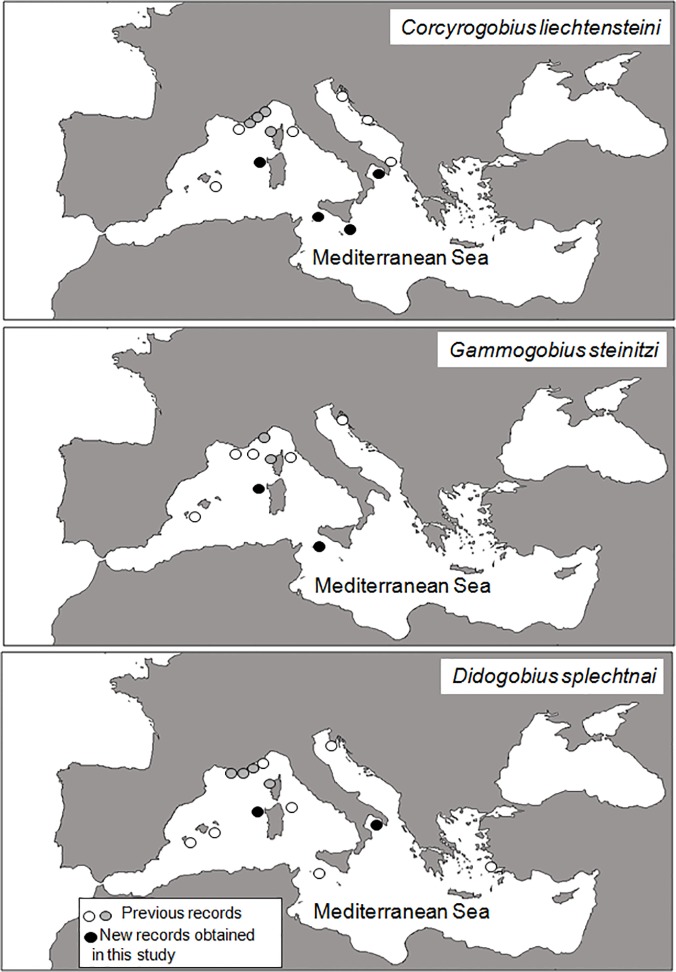
Previous records of cryptobenthic gobids in the Mediterranean (published: white circles; unpublished: grey circles) and new records obtained in this study (black circle).

In this study, we privileged the inclusion of the environmental variability of submerged marine caves (particularly high at the Capo Caccia MPA) so to maximize gathering information on assemblage diversity. The study, conducted at several locations from which the previous information was quite scarce or even missing, thus allowed to collect interesting and new data on the biogeographic distribution of several species. Some speleophilic gobids have been reported for the first time in some localities where they were not previously reported (Fig 5). To our knowledge *Corcyrogobius liechtensteini*, for example, was previously reported only in some localities in Croatia [14], [43], Italy (SE Apulia, [16]; Giglio Island, [44]), Spain (Balearic Island [13], [45] and France (near Marseille, [46]; Port-Cros, Scandola, Cap Roux and Nice, P. Francour, unpublished data). In this study, this species has been reported in all MPAs investigated, which confirms the widespread distribution of this fish inside marine caves across the Mediterranean. *Gammogobius steinitzi* was previously found in Croatia [47], Giglio Island (Italy, [44]), Ibiza island (Spain, [48]), around Marseille and Bagaud Island (France; [46]; [49]), Scandola (Corsica) and Monaco (P. Francour unpublished data). In this study, new records (Capo Caccia and Lampedusa) of *G*. *steinitzi* confirm its basin-wide distribution range. *Didogobius splechtnai* was previously observed in Spain at Ibiza [13] and Cabrera [50], in Italy at Lampedusa [51], Elba and Tavolara islands, in Istria (Croatia; [25]), in various places along the French coast [52], in Corsica (P. Francour unpublished data), and along the southern Aegean Sea coast of Turkey [53]. This study yields new records from Capo Caccia and Porto Cesaro MPAs in Italy.

This study allowed us to census all these gobids, which leads to consider them as common species of cave habitats in the Mediterranean. Patzner [24] stressed that many gobids from the Mediterranean are rarely observed, but also that their rarity is likely to be more apparent than real, due to their small size, economic insignificance and cryptobenthic behavior. In common with many marine taxonomic groups (see [54]), the scarcity of experts capable of identifying small gobies is considered to be another possible reason for interpreting the scarcity of such records [55].

Concerning fish assemblages, this study shows that those associated with marine caves are clearly different from those associated with rocky reefs outside. This evidence is consistent with other more local studies (e.g. [10]). Due to the wider spatial scale of this study, such an evidence can be generalized to a large spatial scale.

The fact that marine caves host different fish assemblages (with some species typical of caves) has never been considered until now in biodiversity/scientific programs aimed, for example, at refining species inventories to maximize fish biodiversity representativity and protection within MPAs in the Mediterranean sea [56], [57]. This shows that until now the contribution of marine caves to local diversity has been substantially neglected. Several fish species that are quite easily detected during daylight hours inside caves (e.g. *Conger conger*, *Phycis phycis*, *Scorpaena notata*) can be hardly detected in rocky reefs outside, because of their nocturnal activity rhythm or because they have a cryptobenthic life style [58], [59]. The cryptobenthic gobid *Didogobius splechtnai*, for example, that in our study was observed only inside caves, is reported to be closely associated with holes and cavities on coralligenous formations [24], on mud in deep waters [45], on pits covered with gravel [51] and among the rhizomes of *Posidonia oceanica* beds [25]. In short, therefore, failure to include caves in monitoring will result in an incomplete census of the fish species present.

Another significant finding of this study is that the structural characteristics of marine caves (i.e. variety of cave sub-habitats such as walls, ceilings, bottoms, etc) may significantly affect the species richness and fish assemblage structures inside. Bussotti and Guidetti [10] reported that the structure of fish assemblages changes along the cave axis in tunnel-like caves, from the entrances to the inner portions. Results of this study show that other cave features may be as much important for shaping fish assemblages inside the caves. In accordance with observations made by Bori *et al*. [60], for example, the complete darkness in the inner cave portions was a condition necessary, but not sufficient to host the strictly speleophilic fish *Grammonus ater*. This point is reinforced by the observations done in an artificial habitat in Monaco (P. Francour; unpublished data): in the inner parts of totally dark concrete dykes, the main cryptic species were *C*. *liechtensteini*, *G*. *steinitzi* and *G*. *ater*. The nature of the substrate of the cave bottom (e.g. rocky or sandy) also plays an important role in influencing the presence of some species and the assemblage structure: when the nature of the bottom is rocky, then fish assemblages were similar to those associated with the cave walls. A similar response to substrate type and features has been recorded elsewhere, from tropical (e.g. [61]) to temperate systems (e.g. [62]), as well as in the Mediterranean Sea [63].

This study suggests that it is fairly difficult to define a ‘typical’ cave fish assemblage or to quantify the distribution patterns in general terms, as fish assemblages may change significantly from cave to cave. Generalizations concerning distribution patterns of fish assemblages can be made in finer terms by selecting a subset of comparable caves in a certain stretch of coast [10], [15], [16]. A similar approach, however, carries the risk to lose information about those caves that have different characteristics from those chosen *a priori* for the sake of formal comparisons (i.e. while using complex experimental designs; Underwood, 1997). In Bussotti *et al*. [15], [16], and Bussotti and Guidetti [10], for example, only blind caves were selected, to specifically aim at testing whether the ‘distance from the entrance’ (and related gradients of light intensity, water motion, etc) was a relevant factor influencing fish distribution inside.

Another important consideration on the results of this study concerns the biodiversity and the related possible conservation measures to implement. It is worth noting the presence in some caves of fish species included in the Red List IUCN [64], such as the dusky grouper *Epinephelus marginatus* and the thorny stingray *Dasyatis centroura* (present exclusively in the Lampedusa MPA), or in the annex III of the Barcelona Convention, such as the brown meagre *Sciaena umbra*. Their presence and frequency within cave habitats can be a valuable tool to support the inclusion of these environments inside MPAs. Some Gobiidae species closely related to marine caves (such as *C*. *liechtensteini*, *D*. *splechtnai*, *G*. *steinitzi*, *O*. *balearica*, *S*. *trigloides*), on the contrary, are generally indicated as DD (‘data deficient’) species in the last assessment of the Red List for the Mediterranean fish. Their association with marine caves, therefore, should be considered in future conservation plans in terms of their contribution to local diversity.

In conclusion, this study allowed to improve and refine the available knowledge on fish diversity in submerged caves in the Mediterranean context. In particular, due to the overall diversity they support, the presence of valuable species for conservation and the ecological role they may fulfill (all this showed in the present and previous studies), submerged marine caves should be prioritized for inclusion within future MPAs and into larger scale marine spatial management plans through the Mediterranean Sea.

## Supporting Information

S1 Dataset Densities of fish detected from each cave and outside rocky reefs. (XLS)

S1 Fig Distance based redundancy analysis (DbRDA) plot of multivariate data constrained to transect length. (DOCX)

S2 Fig Total fish density of each sample (i.e. transect) against transect length. (DOCX)

S3 Fig Species richness of each sample (i.e. transect) against transect length. (DOCX)

S1 Table Fish species recorded at the four cave sub-habitats and outside rocky reefs investigated in this study. (DOC)
