# Supplementary material for: Fish Assemblages of Mediterranean Marine Caves
Source: PLoS One. 2015 Apr 13;10(4):e0122632. doi: 10.1371/journal.pone.0122632 (PMC4395268; doi:10.1371/journal.pone.0122632)
Supplement: S1 Fig — (DOCX) [file pone.0122632.s002.docx]

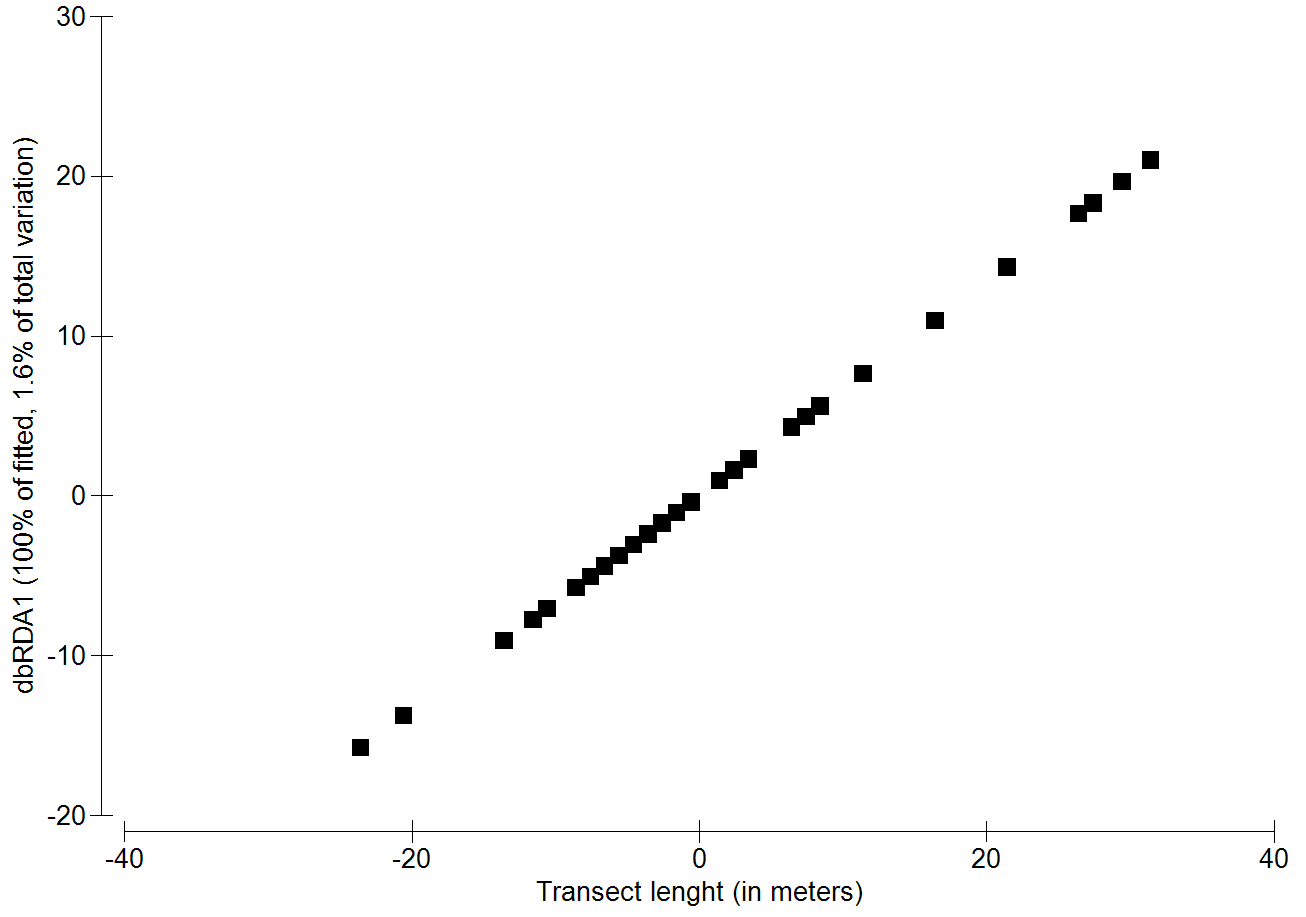


**S1 Fig.** Distance based redundancy analysis (DbRDA) plot of multivariate data constrained to transect length. No significant relationship among transect length and multivariate data of fish assemblage (i.e. in terms of multivariate densities) was detected by DISTLM (n=94, pseudo-f: 1.51, p= 0.13).
