# Supplementary material for: Fish Assemblages of Mediterranean Marine Caves
Source: PLoS One. 2015 Apr 13;10(4):e0122632. doi: 10.1371/journal.pone.0122632 (PMC4395268; doi:10.1371/journal.pone.0122632)
Supplement: S2 Fig — (DOCX) [file pone.0122632.s003.docx]

**S2 Fig.** Total fish density of each sample (i.e. transect) against transect length. Linear fit was not significant. No significant relationship was highlighted between total fish density and transect length (n=94, pseudo-f: 2.49, p= 0.087).
