# Supplementary material for: Fish Assemblages of Mediterranean Marine Caves
Source: PLoS One. 2015 Apr 13;10(4):e0122632. doi: 10.1371/journal.pone.0122632 (PMC4395268; doi:10.1371/journal.pone.0122632)
Supplement: S3 Fig — (DOCX) [file pone.0122632.s004.docx]

**S3 Fig.** Species richness of each sample (i.e. transect) against transect length. Linear fit was not significant. No significant relationship was highlighted between species richness and transect length (n=94, pseudo-f: 0.80, p= 0.43).
