# Supplementary material for: Fish Assemblages of Mediterranean Marine Caves
Source: PLoS One. 2015 Apr 13;10(4):e0122632. doi: 10.1371/journal.pone.0122632 (PMC4395268; doi:10.1371/journal.pone.0122632)
Supplement: S1 Table — (DOC) [file pone.0122632.s005.doc]

**S1 Table.** Fish species recorded at the four cave sub-habitats and outside rocky reefs investigated in this study.

| Family |  |  |  |  |  |
| --- | --- | --- | --- | --- | --- |
| *Species* | Bottom | Ceiling | End | Wall | Outside |
| Apogonidae |  |  |  |  |  |
| Apogon imberbis | X | X | X | X | X |
| Blenniidae |  |  |  |  |  |
| Microlypophris nigriceps | X | X |  | X |  |
| Parablennius gattoruggine |  |  |  |  | X |
| Parablennius rouxi |  |  |  | X | X |
| Parablennius tentacularis |  |  |  | X |  |
| Parablennius zvonimiri |  |  |  | X | X |
| Bothidae |  |  |  |  |  |
| *Bothus podas* | X |  |  |  |  |
| Brotulidae |  |  |  |  |  |
| Grammonus ater |  | X |  | X |  |
| Carangidae |  |  |  |  |  |
| Lichia amia |  |  |  |  | X |
| Seriola dumerili |  |  |  |  | X |
| Trachurus trachurus |  |  |  | X |  |
| Congridae |  |  |  |  |  |
| Conger conger |  | X |  | X |  |
| Centracanthidae |  |  |  |  |  |
| Spicara maena |  |  |  | X | X |
| Dasyatidae |  |  |  |  |  |
| Dasyatis centroura |  |  |  | X |  |
| Gadidae |  |  |  |  |  |
| Trisopterus capelanus |  |  |  | X |  |
| Gobiidae |  |  |  |  |  |
| Corcyrogobius liechtensteini |  | X |  | X |  |
| Didogobius splechtnai |  |  | X | X |  |
| Gammogobius steinitzi |  | X | X | X |  |
| Gobius bucchichi |  |  |  |  | X |
| Gobius cruentatus |  |  |  | X |  |
| Gobius geniporus |  |  |  |  | X |
| Gobius vittatus |  |  |  |  | X |
| Thorogobius ephippiatus | X | X | X | X |  |
| Labridae |  |  |  |  |  |
| Coris julis | X |  | X | X | X |
| *Symphodus doderleini* |  |  |  |  | X |
| *Symphodus mediterraneus* |  |  |  |  | X |
| *Symphodus melanocercus* |  |  |  |  | X |
| *Symphodus ocellatus* |  |  |  |  | X |
| *Symphodus rostratus* |  |  |  |  | X |
| *Symphodus tinca* |  |  |  |  | X |
| Thalassoma pavo |  |  |  |  | X |
| Mugilidae |  |  |  |  | X |
| Mullidae |  |  |  |  |  |
| Mullus surmuletus | X |  | X | X | X |
| Muraenidae |  |  |  |  |  |
| Muraena helena |  |  |  | X | X |
| Ophidiidae |  |  |  |  |  |
| Ophidion barbatum | X |  |  | X |  |
| Phycidae |  |  |  |  |  |
| Phycis phycis | X |  | X | X |  |
| Pomacentridae |  |  |  |  |  |
| Chromis chromis |  | X |  | X | X |
| Scaridae |  |  |  |  |  |
| Sparisoma cretense |  |  |  |  | X |
| Scorpaenidae |  |  |  |  |  |
| Scorpaena maderensis |  | X |  |  | X |
| Scorpaena notata | X | X | X | X | X |
| Scorpaena porcus |  |  |  |  | X |
| Scorpaena scrofa |  |  |  | X |  |
| Serranidae |  |  |  |  |  |
| Anthias anthias | X |  |  |  |  |
| Epinephelus costae |  |  |  | X | X |
| *Epinephelus marginatus* |  |  |  | X | X |
| *Serranus cabrilla* | X | X | X | X | X |
| *Serranus scriba* |  | X |  | X | X |
| Sciaenidae |  |  |  |  |  |
| *Sciaena* *umbra* | X |  |  | X |  |
| Siganidae |  |  |  |  |  |
| *Siganus luridus* |  |  |  |  | X |
| Sparidae |  |  |  |  |  |
| Boops boops |  |  |  | X | X |
| Diplodus annularis |  |  |  | X | X |
| *Diplodus puntazzo* |  |  |  | X | X |
| *Diplodus sargus* |  |  |  | X | X |
| *Diplodus vulgaris* | X |  | X | X | X |
| *Oblada melanura* | X |  |  | X | X |
| *Sarpa salpa* |  |  |  |  | X |
| *Spondyliosoma cantharus* |  |  |  |  | X |
| Sphyraenidae |  |  |  |  |  |
| *Sphyraena* *viridensis* |  |  |  |  | X |
| Tripterygiidae |  |  |  |  |  |
| *Tripterygion delaisi* |  |  |  |  | X |
